# Effect of Flaxseed Intervention on Inflammatory Marker C-Reactive Protein: A Systematic Review and Meta-Analysis of Randomized Controlled Trials

**DOI:** 10.3390/nu8030136

**Published:** 2016-03-04

**Authors:** Guan-Yu Ren, Chun-Yang Chen, Guo-Chong Chen, Wei-Guo Chen, An Pan, Chen-Wei Pan, Yong-Hong Zhang, Li-Qiang Qin, Li-Hua Chen

**Affiliations:** 1 Jiangsu Key Laboratory of Preventive and Translational Medicine for Geriatric Diseases, Department of Nutrition and Food Hygiene, School of Public Health, Soochow University, 199 Renai Road, Dushu Lake Higher Education Town, Suzhou 215123, China; 15850177665@163.com (G.-Y.R.); chen20154232027@126.com (C.-Y.C.); lsguorong@126.com (G.-C.C.); chwpan@suda.edu.cn (C.-W.P.); yhzhang@suda.edu.cn (Y.-H.Z.); qinliqiang@suda.edu.cn (L.-Q.Q.); 2 Department of Urology, The First Affiliated Hospital of Soochow University, Soochow University, 188 Shizi street, Suzhou 215006, China; 15312172967@163.com; 3 Department of Epidemiology and Biostatistics, MOE Key Lab of Environment and Health, School of Public Health, Tongji Medical College, Huazhong University of Science and Technology, 13 Hangkong Road, Wuhan 430030, China; panan@hust.edu.cn

**Keywords:** flaxseed, C-reactive protein, randomized controlled trials, meta-analysis

## Abstract

Functional food-flaxseed and its derivatives (flaxseed oil or lignans) are beneficial for human health, possibly because of their anti-inflammatory effects. C-reactive protein (CRP), a sensitive marker of inflammation was chosen to evaluate the anti-inflammatory effects of flaxseed. We searched randomized controlled trials from PubMed and the Cochrane Library in October 2015 and conducted a meta-analysis to evaluate the effectiveness of flaxseed and its derivatives on CRP. The mean differences (net change) in CRP (mg/L) concentrations were pooled with a random- or a fixed-effects model depending on the results of heterogeneity tests. Overall, flaxseed interventions had no effects on reduction of CRP (*p* = 0.428). The null effects were consistent in the subgroup analysis with multiple studies and population characteristics. Significant heterogeneity was observed in most of the analyses. Meta-regression identified baseline body mass index (BMI) as a significant source of heterogeneity (*P*-interaction = 0.032), with a significant reduction in CRP of 0.83 mg/L (95% confidence interval −1.34 to −0.31; *p* = 0.002) among subjects with a BMI of ≥30 kg/m^2^. In conclusion, our meta-analysis did not find sufficient evidence that flaxseed and its derivatives have a beneficial effect on reducing circulating CRP. However, they may significantly reduce CRP in obese populations.

## 1. Introduction

Cardiovascular disease (CVD) is the leading cause of mortality worldwide [1]. A growing body of evidence suggests that inflammation is a key feature in CVD and its risk factors, such as abdominal obesity, metabolic syndrome and type 2 diabetes (T2D) [2]. The changes in the degree of inflammation could be reflected in the fluctuations in the levels of C-reactive protein (CRP), a sensitive marker of inflammation. According to meta-analyses of long-term prospective studies, increased levels of inflammatory marker CRP were strongly positively associated with CVD risk [3,4]. Several dietary factors, such as plant sterols, fiber, isoflavones and omega-3 (*n*-3) polyunsaturated fatty acids (PUFAs, including eicosapentaenoic acid (EPA), docosahexaenoic acid (DHA), and a-linolenic acid (ALA)) have been shown to be cardiovascular protective based on accumulating evidence from randomized controlled trials (RCTs) [5,6,7]. Reducing inflammation has been recognized as one of the numerous mechanisms by which they may reduce the risk of CVD [6,8]. Flaxseed (linseed, Linum usitatissimum), an edible oil seed/grain, is emerging as an attractive functional food with the richest plant source of ALA (50%–62% of flaxseed oil, or 22% of whole flaxseed) and lignans (a class of phytoestrogen, range: 0.2–13.3 mg/g flaxseed) [9,10,11]. In addition, flaxseed contains abundant dietary fiber (28% by weight), a third of which is soluble fiber [9,10,11].

ALA has been shown to have anti-inflammatory effects through the downregulation of the expression of hepatic inflammatory genes in diabetic rats and a remarkable reduction of plasma CRP levels in rats fed with a high-fat diet [12,13]. Whole flaxseed supplementation has been shown to mitigate pathophysiology of atherosclerosis in myocardial ischemia rat models through decreasing levels of inflammatory markers [14].

The special composition of flaxseed and its possible mechanisms in CVD protection inspire many researchers to perform clinical trials to determine the outcomes of flaxseed intervention (whole flaxseed, flaxseed oil, or lignans) on various cardiovascular risk factors, particularly inflammation status reflected in inflammatory marker levels of CRP [11,15,16,17,18,19,20,21,22,23,24,25,26,27,28,29,30,31,32,33]. However, the findings from those clinical trials were inconsistent, and the discrepancies may be attributable to aspects of the study design (e.g., study power, the patient recruited, sample size, study duration, flaxseed or its derivatives dose used). Therefore, we performed a meta-analysis of published RCTs to evaluate whether administration of flaxseed or its derivatives could ameliorate inflammation status.

## 2. Methods

### 2.1. Literature Search

We made attempts to follow the Preferred Reporting Items for Systematic Reviews and Meta-Analysis (PRISMA) guidelines in the report of this meta-analysis and registered our report in PROSPERO (International prospective register of systemic reviews; CRD42016034184) [34]. Two researchers (G.-Y.R. and C.-Y.C) independently searched PubMed and the Cochrane Library for English-language reports of clinical trials published in October 2015. Studies describing the effects of flaxseed or its derivatives (in the form of whole or ground flaxseed, flaxseed oil, or lignan supplement) in adults were selected. The following keywords were applied to the search: (“C-reactive protein” OR CRP OR inflammation) AND (flax* OR linseed OR lignan OR “Linum usitatissimum”). For literature search in PubMed, the filters, such as Full text, Humans and English were activated. The search in the Cochrane Library was limited to Trials (the detailed search strategy is shown in Table S1). Additionally, the reference lists of selected studies and relevant reviews [10,35,36,37,38,39] were also checked to ensure a complete collection. Attempts were also made to contact investigators for unpublished data.

### 2.2. Study Selection

Studies were included for analysis if they met the following criteria: (1) The subjects consumed flaxseed or its derivatives for at least 2 weeks; (2) the study was an RCT with either a parallel or crossover design; (3) the study reported the dose of flaxseed, lignans, or flaxseed oil (or ALA); and (4) the effects of flaxseed or its derivatives on inflammatory marker CRP could be extracted from the report.

### 2.3. Data Extraction and Quality Assessment

The following information was extracted and tabulated for analysis: authors, publication year, sample size and attrition, dose and type of the intervention, control treatment, study duration, study design (crossover or parallel), participant information (sex, age, health status), and CRP data. The study quality was assessed with the Jadad score (randomization, blinding, description of withdrawals and dropouts, methods of randomization, and double-blinding status) for RCTs [40]. The total score was the sum of the 5 points, which generated a scale from 0 to 5; higher numbers represented better quality.

### 2.4. Statistical Analysis

The effect was defined as the mean difference (net change) in CRP (mg/L) concentrations. For parallel trials, the net changes were calculated using the difference (intervention minus control) of the changes (final values minus baseline values) of the mean values. For cross-over trials, the net changes were calculated as the difference of mean values at the end of the intervention and control periods. Where necessary, standard errors and confidence intervals (CIs) were converted to standard deviations (SDs) for the analysis. The SDs for changes from baseline in the intervention and control groups were obtained. If not specified in the original paper, we computed the missing SDs using the method proposed by Follmann *et al.* [41], where a correlation coefficient of 0.5 was assumed.

The heterogeneity of the effect sizes among studies was tested using the *I*^2^ and the Cochrane *Q* tests statistics. Either a fixed-effect or random-effects model (in the presence of heterogeneity, *p* value for *Q* test < 0.1 or *I*^2^ > 50%) was used to calculate the combined effect size. The influence of a single study on the overall effect estimate was investigated by omitting one study in each turn. Meta-regression was implemented to examine characteristics of the studies that were hypothesized as influencing the observed treatment effects. We further conducted pre-specified subgroup analysis stratified by type of intervention (whole flaxseed, flaxseed oil or flaxseed lignan), intervention dose, study duration, study design, study sample size, study quality score (measured with the Jadad score), participant gender, participant BMI, participant age, and baseline CRP level. Publication bias was evaluated with the Funnel plots and Egger’s regression model.

All analyses were performed using STATA version 11.0 (StataCorp, College Station, TX, USA). *p* < 0.05 was deemed statistically significant, except where otherwise specified.

## 3. Results

### 3.1. Study Selection

**Figure 1:**
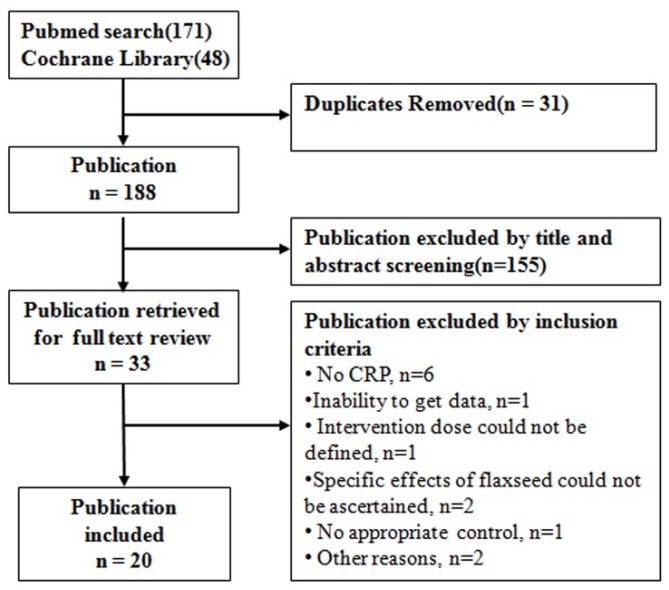
Flow chart of study selection.

A total of 219 citations (171 items from PubMed and 48 items from the Cochrane Library) was yielded from the literature search (search strategy in Table S1). After the title and abstract screening, 33 items were retrieved for more detailed reviews. A total of 13 reports were excluded after carefully reading the full reports (see Table S2). Finally, we identified 20 studies that were suitable for our meta-analysis (Figure 1).

### 3.2. Characteristics of the Studies

The primary characteristics of these 20 studies are outlined in Table 1. Overall, 1378 subjects were randomly assigned in these trials, and 1213 (88%) participants completed the studies. The mean age of the participants ranged from 25.6 to 65 years old. Among the 20 trials, 5 were conducted exclusively on women [15,17,20,21,32], 2 were on men [16,30], and the other 13 were on both genders (1 trial did not indicate the gender composition [28]).

Flaxseed in whole [16,17], ground [11,22,24,31,33] or flour [18,19] form was tested in 9 trials with doses from 13.0 to 60.0 g/day (median: 30.0 g/day). Wheat, wheat bran/germ, or manioc flour were employed as the control regimen in these studies. However, in one study [16], participants in the control arm were allocated to low-fat diets, whereas participants in the intervention arm received a low-fat diet plus additional flaxseed (30 g/day). In another study [11], all the participants in both control and intervention group were asked to follow low-fat, low-cholesterol diets. Flaxseed oil has been tested in another 8 trials [20,23,25,26,27,28,30,32], with doses ranging from 1.0 to 11.6 g/day for ALA (median: 5.65 g/day). The control regimens included oils enriched in the monounsaturated fatty acids (MUFAs) olive oil [20,25] or in the polyunsaturated omega-6 fatty acid safflower, sunflower oil, or soybean oil [23,28,30,32]. In the remaining 3 trials [15,21,29], flaxseed lignan supplement was used and the doses were 360, 500, and 600 mg/day, respectively. The controls were assigned to placebo.

The trials varied in length from 2 to 52 weeks, with a median duration of 12 weeks. Most of the trials (13 trials) adopted a parallel study design [11,16,17,19,20,22,23,24,26,27,28,30,33], whereas the other 7 trials used a crossover design [15,18,21,25,29,31,32]. In most studies, the participants were instructed to maintain their dietary habits, except the two low-fat trials [11,16]. The investigators attempted to provide similar amounts of total fat and saturated fat in both the intervention and control arms.

### 3.3. Changes in CRP Concentration

**Figure 2:**
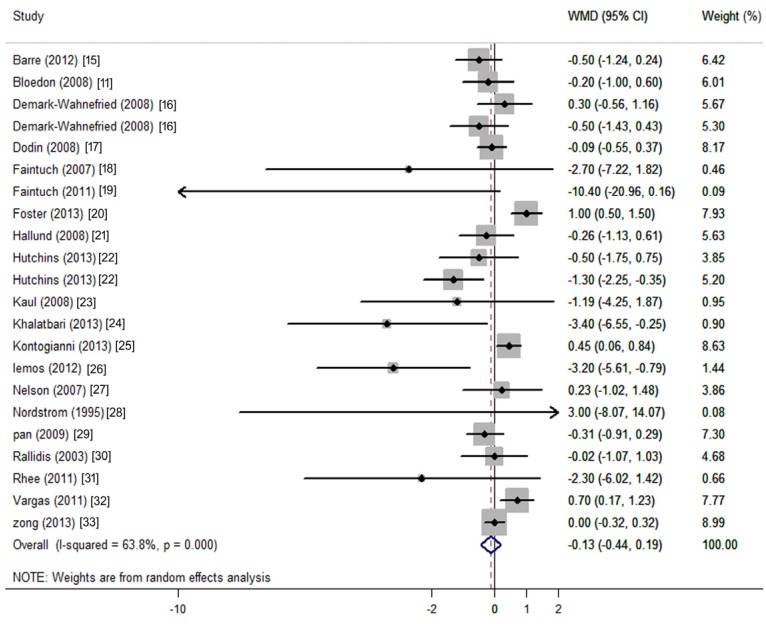
Meta-analysis of flaxseed intervention on net changes (95% CI) of CRP. CI: confidence interval. WMD: weighted mean difference; The horizontal lines denote the 95% CIs: some of which extend beyond the limits of the scales. The square represents the point estimate of each study. The diamond represents the overall pooled estimate of the treatment effect.

The net changes and corresponding 95% CIs for CRP (22 comparisons from 20 studies) was presented in Figure 2. Flaxseed or its derivatives non-significantly changed CRP (−0.13 mg/L; 95% CI: −0.44 to 0.19; *p* = *0.428*; Figure 2). Because the test for heterogeneity was significant for CRP (*I*^2^ = 63.8%, *p* < 0.001), we reported the results from the random-effects model.

### 3.4. Subgroup and Meta-Regression Analysis for CRP

Considering that the basal levels of CRP, type of study design (parallel or crossover design), study quality (measured with the Jadad score), study duration, sex composition, age, body mass index (BMI), type of intervention (whole flaxseed, flaxseed oil, or lignan supplement), and intervention dose may influence the net changes of CRP, we conducted meta-regression analysis based on these variables. We detected sources of heterogeneity according to multiple pre-defined study and population characteristics and found that the type of intervention (*p* = 0.008), baseline BMI (*p* = 0.032), and possibly baseline CRP (*p* = 0.064) contributed to the heterogeneity among studies, but only the stratum of obese populations showed significant results in CRP reduction (−0.83 mg/L; 95% CI: −1.34 to −0.31; *p* = 0.002) (Table 2). Among the strata of whole flaxseed intervention, lignan intervention and participants with higher BMI showed non-significant tendencies toward reduced CRP, which may be due to a low statistical power resulting from small effect of flaxseed and/or small sample size included (Table 2).

### 3.5. Publication Bias

**Figure 3:**
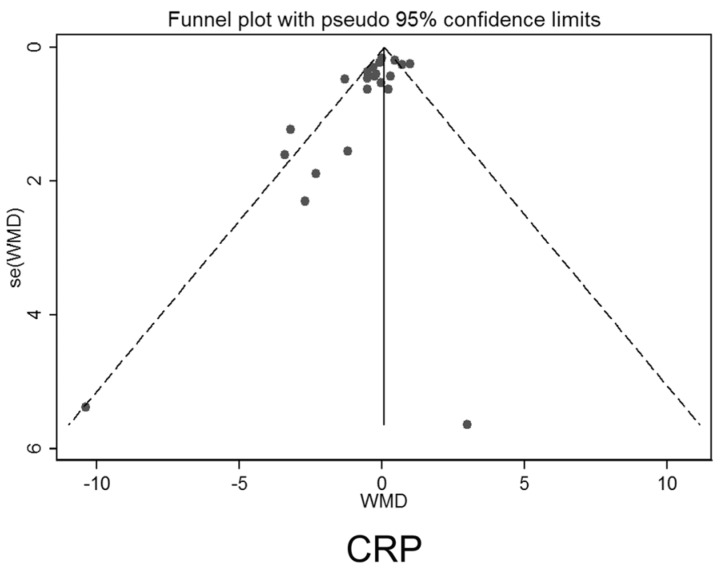
Funnel plots for testing publication bias.

Both the funnel plot and Egger’s test (*p =* 0.007) showed evidence of publication bias in CRP (Figure 3).

## 4. Discussion

This meta-analysis provides evidence of no general benefit of flaxseed or its derivatives supplementation on decreasing CRP levels. However, the supplementation significantly decreased CRP levels in studies, where the recruited participants’ BMI were over 30 kg/m^2^.

Many studies have repeatedly shown that obese [42] or elderly [43] people tend to have higher blood levels of CRP, and people with chronically elevated levels of inflammation may be most likely to benefit from this intervention [44]. This has been confirmed in the subgroup analysis that CPR level was significantly reduced in studies with mean BMI over 30 kg/m^2^ (Table 2). Additionally, we found a trend toward a greater reduction of CPR in age over 50 years (Table 2). Three of the included studies needed to be mentioned individually. The participants recruited by Faintuch *et al.* [18,19] were morbidly obese with BMIs over 40 kg/m^2^. The participants recruited by Nordstrom *et al.* [28] were subjects with rheumatoid arthritis. CRP levels were over 10 mg/L in these 3 studies. Clinically, CRP over 10 mg/L is suggestive of active inflammation or infection [45]. Because the SDs of the net change of CRP were large and sample sizes were very small (around 10 in each group), the weight of these 3 studies is negligible (less than 1%). An exclusion of these 3 studies from the meta-analysis did not significantly change the pooled results (Table 2). Whether flaxseed intervention could decrease CRP levels in these active inflammatory situations needs to be further studied.

Intake of *n*-3 PUFAs (EPA and DHA) has been shown to have beneficial effects on CVD, diabetes, and other obesity-related diseases [46]. A recent meta-analysis showed that marine-derived *n*-3 PUFAs supplementation had a significant lowering effect on CRP levels [47]. However, it is unclear whether ALA, as a plant-based source of *n*-3 PUFA, has similar effects on chronic diseases and inflammation. Unlike fish oil, ALA is more readily available and quite inexpensive. Flaxseed was considered as an alternative source of marine-derived omega-3 fatty acid, and some studies were conducted to examine its beneficial role in patients with T2D, rheumatoid arthritis, and chronic hemodialysis [24,32,48]. However, in our current meta-analysis, we showed that flaxseed oil did not have a significant CRP-lowering effect. One of the plausible explanations for this null result is that the conversion from ALA to EPA or DHA was considered negligible (conversion rate has been suggested to be as low as 5%) [49]; thus, the biologically effective dose may not be reached through such a conversion. Another explanation for the null findings is that the effects of flaxseed oil may have been masked by the use of MUFA- or *n*-6 PUFA-enriched oils as the control regimen in these studies. Previous studies have shown that, compared to saturated fatty acids, MUFAs and *n*-6 PUFAs also have an inflammation-lowering effect [50,51]. Whether a replacement of MUFAs or *n*-6 PUFAs for saturated or trans fatty acids as the control regimen could lower CRP remains to be elucidated.

Our findings from the meta-analysis showed that whole flaxseed reduced CRP levels and the statistical result was borderline significant. Besides *n*-3 fatty acid, flaxseed is a good source of dietary fiber (28% by weight), in particular soluble fiber [9,10,11]. Dietary fiber can be partially or completely fermented to short chain fatty acids (acetate, propionate, and butyrate). One study showed that propionate downregulated the pro-inflammatory cytokine TNF-αin adipose tissue [52]. Recently, Jiao *et al.* [53] conducted a meta-analysis and showed that dietary fiber (total fiber intake 8 g/day higher) significantly reduced CRP levels in overweight and obese adults. Thus, this CRP-lowering effect might be due to the fiber component in whole flaxseed. Lignans are a group of polyphenols with antioxidant prosperities and are abundant in flaxseed (range: 0.2–13.3 mg/g) [35]. Although lignans are not classified as dietary fibers, they share some of the chemical characteristics of lignin, which is an insoluble fiber. However, human studies that examine the role of lignans on inflammation are still limited, and whether lignans alone have a CRP-lowering effect needs to be confirmed or refuted.

This meta-analysis included studies that had a relatively high Jadad score. However, it was limited by a potential publication bias, which is revealed by the asymmetry of the funnel plot and the Egger’s model. Publication bias suggests that some small studies with negative findings may have been missed or unpublished. Including these studies would further promote our results to a null effect. In addition, there is considerable heterogeneity across studies, which complicated the interpretation of our findings. Given the variation in study characteristics, it is not surprising that there is substantial heterogeneity among individual studies. The participants in these studies are overweight/obese adults, hemodialysis patients with dyslipidaemia, patients with renal failure, patients with rheumatoid arthritis, T2DM or metabolic syndrome, and adults who are defined “healthy”. A healthy status may influence the effect of flaxseed or its derivatives on inflammatory response differently. The intestinal microbiota composition varies between healthy and diseased individuals [54]. As the components of flaxseed need to be fermented or metabolized by human intestinal bacteria to exert their biological effects, gut microbiota may also change the effect of flaxseed on inflammatory response [38]. Subgroup analysis results indicated a significantly larger reduction of CRP in subjects with BMIs over 30 kg/m^2^. This finding was also supported by meta-regression analysis that BMI was a source of heterogeneity among trials. This result is important because obesity, an established risk factor for CVD, is associated with elevated levels of CRP [55]. A decrease in CRP by flaxseed supplementation is helpful for alleviating the risk of CVD [55]. Although our meta-analysis did not find sufficient evidence that flaxseed and its derivatives have a beneficial effect on reducing circulating CRP in unstratified populations, their CVD prevention role cannot be denied. As is well known, in addition to inflammation, high blood pressure and dyslipidemia are also risk factors of CVD. Recent meta-analysis showed that supplementation with various flaxseed products decreased both systolic blood pressure (BP) and diastolic BP [56]. In additon, a meta-analysis by Pan *et al.* found that flaxseed reduced circulating total and LDL-cholesterol concentrations [7]. The intervention of whole flaxseed reduced total or LDL cholesterol by ≈0.2 mmol/L, which is estimated to have resulted in a reduction of ≈3% in all-cause mortality and of 6% in both coronary heart disease–related mortality and total events [7,57]. Therefore, the effects of flaxseed on CVD prevention appear to be clinically significant. Thus, functional foods/nutraceutials such as garlic [58], green tea [59], astaxanthin [60] and flaxseed [7], *etc*. that have been demonstrated to reduce the risk of CVD, are encouraged to be incorporated into our diet, as they might reduce the drug dose, such as statins, the commonly prescribed drugs to prevent cardiovascular events or drug-related side effects as an adjuvant therapy [61]. Our data suggest that future well-designed studies with large sample sizes and adequate durations are needed to investigate the effectiveness of whole flaxseed on inflammatory factors amelioration, particularly in obese populations.

## 5. Conclusions

Our meta-analysis did not find sufficient evidence that flaxseed and its derivatives have a beneficial effect on reducing circulating CRP. However, the supplementation of flaxseed and its derivatives may significantly reduce CRP in obese populations.

## Figures and Tables

**Table 1:** Characteristics of the 20 included studies, with 22 comparisons.

Author, Publication Year and Reference Number	Enroll	Comp	Mean Age	Mean BMI	Sex Male	Intervention	WF	ALA	LIG	Duration	Patient Features	Jadad Score	Design	Diet Type
	*n*	*n*	Years		%		g/Day	g/Day	mg/Day					
Barre, 2012 [15]	24	16	62.2	31.2	0	LIG/PLA	NA	NA	600	12	Overweight or obese	3	RC	Usual
Bloedon, 2008 [11]	62	58	56.9	27.8	50	GF/WB	40.0	3.2	640.0	10	Hypercholesterolemia	4	RP	LF LC
Demark-Wahnefried, 2008 [16]	80	71	59.2	28.8	100	WF/Control	30.0	NR	NR	4.5	Prostate cancer	4	RP	Usual
Demark-Wahnefried, 2008 [16]	81	78	59.2	28.8	100	WF/Control	30.0	NR	NR	4.5	Prostate cancer	4	RP	LF
Dodin, 2008 [17]	199	177	54.7	26.2	0	WF/WG	40.0	9.1	21.0	52	Healthy	5	RP	Usual
Faintuch, 2007 [18]	24	24	40.8	47.1	17	FF/MF	30.0	5.0	NR	2	Obese	4	RC	Usual
Faintuch, 2011 [19]	20	18	46.3	44.9	17	FF/CF	60.0	10.0	NR	12	Obese	4	RP	Usual
Foster, 2013 [20]	20	20	65.0	28.6	0	FXO/OO	NA	1.2	NA	12	T2DM	4	RP	Usual
Hallund, 2008 [21]	23	22	61.0	24.1	0	LIG/PLA	NA	NA	500.0	6	Healthy	4	RC	Usual
Hutchins, 2013 [22]	41	25	58.6	30.4	44	GF/0	13.0	2.9	59.0	12	Overweight or obese	3	RP	Usual
Hutchins, 2013 [22]	41	25	58.6	30.4	44	GF/0	26.0	5.8	118.0	12	Overweight or obese	3	RP	Usual
Kaul, 2008 [23]	100	88	33.7	24.7	38	FXO/SFO	NA	1.0	NA	12	Healthy	3	RP	Usual
Khalatbari, 2013 [24]	30	30	54.2	26.3	53	GF/Control	40.0	NR	NR	8	Hemodialysis patients	3	RP	Usual
Kontogianni, 2013 [25]	53	37	25.6	21.9	21	FXO/OO	NA	8.0	NA	6	Healthy	2	RC	Usual
Lemos, 2012 [26]	160	114	57.0	24.7	58	FXO/MO	NA	2.0	NA	17	Renal failure	3	RP	Usual
Nelson, 2007 [27]	56	51	38.0	29.8	21	FXO/Control	NA	11.6	NA	8	Overweight or obese	2	RP	Usual
Nordstrom, 1995 [28]	22	22	52	NR	NR	FXO/SAO	NA	9.6	NA	12	Rheumatoid arthritis	3	RP	Usual
Pan, 2009 [29]	73	70	64	25.0	37	LIG/PLA	NA	NA	360.0	12	T2DM	5	RC	Usual
Rallidis, 2003 [30]	76	76	51.2	28.2	100	FXO/SAO	NA	8.0	NA	12	Dyslipidaemic patients	3	RP	Usual
Rhee, 2011 [31]	11	9	54.7	31.4	44	GF/WB	40	NR	NR	12	Obese and glucose intolerant people	2	RC	Usual
Vargas, 2011 [32]	34	34	34.1	29.2	0	FXO/SOY	NA	3.3	NA	6	Polycystic ovary syndrome	3	RC	Usual
Zong, 2013 [33]	189	173	48.8	25.3	56	GF/0	30	7.0	NR	12	Metabolic syndrome	5	RP	Usual

ALA: α-linolenic acid; BMI: body mass index; CF: cassava flour; Comp: completer; Enroll: enrollment; FF: flaxseed flour; FXO: flaxseed oil; GF: ground flaxseed; LC: low cholesterol; LF: low fat; LIG: lignans; MF: manioc flour; NA: not applicable; NR: not reported; OO: olive oil; PLA: placebo; RC: randomized crossover design; RP: randomized parallel design; SAO: safflower oil; Soyb: soybean oil; SFO: sunflower oil; T2DM: type 2 diabetes mellitus; WB: wheat bran; WF: whole flaxseed; WG: wheat germ.

**Table 2:** Pooled estimates of effects on CRP within various subgroups.

Subgroups	No. of Comparisons	Net Change (95% CI)	*p* for Interaction	*I^2^*	*p* for Heterogeneity	Analysis Models
**Type of intervention**						
Whole flaxseed	11	−0.35 (−0.75, 0.05)	0.008	46.3%	0.045	Random-effect
Flaxseed oil	8	0.39 (−0.09, 0.87)		55.6%	0.027	Random-effect
Lignan	3	−0.36 (−0.77, 0.05)		0.00%	0.089	Fixed-effect
**Dose of whole flaxseed**						
<40 g	6	−0.34 (−0.85, 0.17)	0.258	47.9%	0.087	Random-effect
≥40 g	5	−0.63 (−1.62, 0.36)		55.4%	0.062	Random-effect
**CRP**						
<10 mg/L	19	−0.10 (−0.41, 0.21)	0.064	65.7%	<0.0001	Random-effect
≥10 mg/L	3	−3.04 (−6.93, 0.85)		34.1%	0.220	Fixed-effect
**BMI**						
<30 kg/m^2^	15	0.06 (−0.25, 0.37)	0.032	63.6%	<0.0001	Random-effect
≥30 kg/m^2^	6	−0.83 (−1.34, −0.31)		21.8%	0.270	Fixed-effect
**Gender**						
Female	4	0.30 (−0.34, 0.95)	0.191	82.1%	0.001	Random-effect
Male	3	−0.06 (−0.60, 0.49)		0.00%	0.464	Fixed-effect
Both	15	−0.39 (−0.82, 0.04)		57.6%	0.003	Random-effect
**Study design**						
Crossover	7	0.01 (−0.47, 0.49)	0.638	65.9%	0.015	Random-effect
Parallel	15	−0.23 (−0.66, 0.20)		61.9%	<0.0001	Random-effect
**Study duration**						
<12 weeks	9	0.09 (−0.31, 0.50)	0.649	48.9%	0.048	Random-effect
≥12 weeks	13	−0.31 (−0.77, 0.16)		69.1%	<0.0001	Random-effect
**Sample size**						
<50	10	−0.20 (−0.99, 0.58)	0.710	69.4%	0.001	Random-effect
≥50	12	−0.15 (−0.45, 0.15)		51.7%	0.019	Random-effect
**Age**						
<50 years	7	0.27 (−0.17, 0.70)	0.268	52.2%	0.051	Random-effect
≥50 years	15	−0.32 (−0.74, 0.10)		66.5%	<0.0001	Random-effect
**Quality Score**						
<4	12	−0.39 (−0.97, 0.20)	0.902	68.1%	<0.0001	Random-effect
≥4	10	−0.32 (−0.37, 0.37)		61.6%	0.005	Random-effect

*p* for interaction: for heterogeneity between subgroups with meta-regression analysis.

## Supplementary Materials

The following are available online at http://www.mdpi.com/2072-6643/8/3/136/s1, Table S1: Literature search strategy for meta-analysis, Table S2: Characteristics of excluded studies and reasons for exclusion.
