# Supplementary material for: Effect of Flaxseed Intervention on Inflammatory Marker C-Reactive Protein: A Systematic Review and Meta-Analysis of Randomized Controlled Trials
Source: Nutrients. 2016 Mar 4;8(3):136. doi: 10.3390/nu8030136 (PMC4808865; doi:10.3390/nu8030136)
Supplement: Supplementary file 1 [file nutrients-08-00136-s001.docx]

Effect of Flaxseed Intervention on Inflammatory Marker C-Reactive Protein: A Systematic Review and Meta-Analysis of Randomized Controlled Trials

Guan-Yu Ren, Chun-Yang Chen, Guo-Chong Chen, Wei-Guo Chen, An Pan,
Chen-Wei Pan, Yong-Hong Zhang, Li-Qiang Qin and Li-Hua Chen

**Table S1.** Literature search strategy for meta-analysis.

| PubMed (Searched October 2015) |
| --- |
| 1 (“C-reactive protein” OR CRP OR inflammation) AND (flax* OR linseed OR lignan OR  “Linum usitatissimum”) |
| 2 Filters activated: Full text, Humans, English |
| Cochrane Library (Searched October 2015) |
| 1 Search by (All text) (“C-reactive protein” OR CRP OR inflammation) AND (flax* OR linseed OR lignan OR “*Linum usitatissimum*”) |
| 2 Search limits: Trials |

**Table S2.** Characteristics of excluded studies and reasons for exclusion.

| **Study** | **Design** | **Reasons for Exclusion** |
| --- | --- | --- |
| Barcelo-Coblijn, 2008 [1] | RP | Inability to get CRP data |
| Baril-Gravel, 2015 [2] | RC | Experimental group was a mix(60% flax oil and 40% safflower oil), the specific effects of flaxseed could not be ascertained |
| Bemelmans, 2004 [3] | RP | ALA-enriched margarines were used *ad libitum*, thus, ALA dose could not be defined. |
| Cornish, 2009 [4] | RP | No results of CRP |
| Cornish, 2009 [5] | RP | No results of CRP |
| Gillingham, 2008 [6] | RP | Experimental group was a blended oil(flax oil and rapeseed oil), the specific effects of flaxseed could not be ascertained |
| Dewell, 2011 [7] |  |  |
| Heymach, 2011 [8] | RP | No results of CRP |
| Paschos, 2005 [9] | RP | No appropriate control |
| Ristic-Medic [10] | Cross-sectional study | Not RCT |
| Thies, 2001 [11] | RP | No results of CRP |
| Wallace, 2003 [12] | RP | Cytokine production by peripheral blood mononuclear cells *in vitro* |
| West, 2010 [13] | RC | No results of CRP |

CRP, C-reactive protein; RC, randomized crossover design; RCT, randomized controlled trials;
RP, randomized parallel design.

References

1. Barcelo-Coblijn, G.; Murphy, E.J.; Othman, R.; Moghadasian, M.H.; Kashour, T.; Friel, J.K. Flaxseed oil and fish-oil capsule consumption alters human red blood cell *n*-3 fatty acid composition: A multiple-dosing trial comparing 2 sources of *n*-3 fatty acid. *Am. J. Clin. Nutr.* 2**008**, *88*, 801–809.
2. Baril-Gravel, L.; Labonte, M.E.; Couture, P.; Vohl, M.C.; Charest, A.; Guay, V.; Jenkins, D.A.; Connelly, P.W.; West, S.; Kris-Etherton, P.M.; *et al*. Docosahexaenoic acid-enriched canola oil increases adiponectin concentrations: A randomized crossover controlled intervention trial. *Nutr. Metab. Cardiovasc. Dis.* 2**015**, *25*, 52–59.
3. Bemelmans, W.J.; Lefrandt, J.D.; Feskens, E.J.; van Haelst, P.L.; Broer, J.; Meyboom-de Jong, B.; May, J.F.; Tervaert, J.W.; Smit, A.J. Increased alpha-linolenic acid intake lowers C-reactive protein, but has no effect on markers of atherosclerosis. *Eur. J. Clin. Nutr.* **2004**, *58*, 1083–1089.

1. Cornish, S.M.; Chilibeck, P.D. Alpha-linolenic acid supplementation and resistance training in older adults. *Appl. Physiol. Nutr. Metab.* **2009**, *34*, 49–59.
2. Cornish, S.M.; Chilibeck, P.D.; Paus-Jennsen, L.; Biem, H.J.; Khozani, T.; Senanayake, V.; Vatanparast, H.; Little, J.P.; Whiting, S.J.; Pahwa, P. A randomized controlled trial of the effects of flaxseed lignan complex on metabolic syndrome composite score and bone mineral in older adults. *Appl. Physiol. Nutr. Metab.* **2009**, *34*, 89–98.
3. Gillingham, L.G.; Gustafson, J.A.; Han, S.Y.; Jassal, D.S.; Jones, P.J. High-oleic rapeseed (canola) and flaxseed oils modulate serum lipids and inflammatory biomarkers in hypercholesterolaemic subjects.
   *Br. J. Nutr.* **2011**, *105*, 417–427.
4. Dewell, A.; Marvasti, F.F.; Harris, W.S.; Tsao, P.; Gardner, C.D. Low- and high-dose plant and marine
   (*n*-3) fatty acids do not affect plasma inflammatory markers in adults with metabolic syndrome. *J. Nutr.* **2011**, *141*, 2166–2171.
5. Heymach, J.V.; Shackleford, T.J.; Tran, H.T.; Yoo, S.Y.; Do, K.A.; Wergin, M.; Saintigny, P.; Vollmer, R.T.; Polascik, T.J.; Snyder, D.C.; *et al*. Effect of low-fat diets on plasma levels of NF-kappaB-regulated inflammatory cytokines and angiogenic factors in men with prostate cancer. *Cancer Prev. Res. (Phila)* **2011**, *4*, 1590–1598.
6. Paschos, G.K.; Yiannakouris, N.; Rallidis, L.S.; Davies, I.; Griffin, B.A.; Panagiotakos, D.B.; Skopouli, F.N.; Votteas, V.; Zampelas, A. Apolipoprotein E genotype in dyslipidemic patients and response of blood lipids and inflammatory markers to alpha-linolenic Acid. *Angiology* **2005**, *56*, 49–60.
7. Ristic-Medic, D.; Perunicic-Pekovic, G.; Rasic-Milutinovic, Z. Effects of dietary milled seed mixture on fatty acid status and inflammatory markers in patients on hemodialysis. *Sci. World J.* **2014**, *2014*, 563576.
8. Thies, F.; Miles, E.A.; Nebe-von-Caron, G.; Powell, J.R.; Hurst, T.L.; Newsholme, E.A.; Calder, P.C. Influence of dietary supplementation with long-chain n-3 or n-6 polyunsaturated fatty acids on blood inflammatory cell populations and functions and on plasma soluble adhesion molecules in healthy adults. *Lipids* **2001**, *36*, 1183–1193.
9. Wallace, F.A.; Miles, E.A.; Calder, P.C. Comparison of the effects of linseed oil and different doses of fish oil on mononuclear cell function in healthy human subjects. *Br. J. Nutr.* **2003**, *89*, 679–689.
10. West, S.G.; Krick, A.L.; Klein, L.C.; Zhao, G.; Wojtowicz, T.F.; McGuiness, M.; Bagshaw, D.M.; Wagner, P.; Ceballos, R.M.; Holub, B.J.; *et al*. Effects of diets high in walnuts and flax oil on hemodynamic responses to stress and vascular endothelial function. *J. Am. Coll. Nutr.* **2010**, *29*, 595–603.
